# Swallowing in Parkinson Patients versus Healthy Controls: Reliability of Measurements in Videofluoroscopy

**DOI:** 10.1155/2011/380682

**Published:** 2011-10-03

**Authors:** Laura W. J. Baijens, Renée Speyer, Valéria Lima Passos, Walmari Pilz, Nel Roodenburg, Pere Clave

**Affiliations:** ^1^Department of Otorhinolaryngology, Head and Neck Surgery, Maastricht University Medical Center, P.O. Box 5800, 6202 AZ Maastricht, The Netherlands; ^2^Department of Speech and Language Pathology, HAN University of Applied Sciences, 6525 EN Nijmegen, The Netherlands; ^3^Department of Methodology and Statistics, Maastricht University, 6229 HA Maastricht, The Netherlands; ^4^Department of Neurology, Maastricht University Medical Center, 6202 AZ Maastricht, The Netherlands; ^5^Departamento de Cirugía, Hospital de Mataróy CIBEREHD, Instituto de Salud Carlos III, Mataró, Spain

## Abstract

*Objective*. To determine and describe the pathophysiological aspects of oropharyngeal swallowing in patients with Parkinson's disease more accurately, a pilot study of qualitative as well as quantitative parameters of swallowing was performed using videofluoroscopy (VFS). *Methods*. Ten patients with a diagnosis of idiopathic Parkinson's disease having dysphagic complaints and ten healthy age- and gender-matched control subjects underwent a standardized videofluoroscopic swallowing protocol. Information on the swallowing function was derived from temporal, spatial, and descriptive visuoperceptual parameters. Intra- and interrater reliability was calculated. *Results*. No significant differences were found between Parkinson patients and healthy control subjects for the majority of the reliable variables. *Conclusions*. It was concluded that swallowing function seemed to be preserved in the early stages of Parkinson's disease. Furthermore, the reliability of many quantitative as well as qualitative swallowing parameters proved insufficient, raising questions about the interpretation of study outcomes in videofluoroscopy.

## 1. Introduction

Dysphagia is a common symptom of Parkinson's disease [1]. In 75–100% of patients with Parkinson's disease, swallowing abnormalities have been observed during videofluoroscopic examination (VFS) [2]. Severe dysphagia can result in malnutrition, dehydration, aspiration pneumonia, and sudden death [3]. Tracheal aspiration is not an uncommon phenomenon in Parkinson patients [2, 4–6]. A substantial number of them do not have subjective complaints of dysphagia, despite the presence of abnormalities observed in the swallowing act, including silent aspiration during VFS examination [2, 6]. The quality of life decreases as the dysphagia progressively affects oral feeding [7, 8]. Previous studies describe oral and pharyngeal phase abnormalities during VFS examination using visuoperceptual parameters, such as piecemeal deglutition, pumping tongue motion, or postswallow vallecular pooling, while others use timed parameters, for example, oropharyngeal transit time, duration of the upper esophageal sphincter opening, or total swallow duration [5, 9, 10]. There is hardly any literature on the pathophysiology of swallowing in patients with Parkinson's disease using spatial or timed parameters in VFS recordings [6].

The purpose of this study is twofold: to determine the reliability of timed as well as spatial parameters in videofluoroscopy of swallowing necessary to interpret the study outcome and to determine the differences in swallowing function between patients with Parkinson's disease and healthy control subjects, matched for age and gender. Qualitative assessment of swallows was based on visuoperceptual evaluation of videofluoroscopic signs by an expert panel, whereas quantitative assessment was obtained by means of a specialized computer software application that allowed capturing and digitizing frames in videofluoroscopic recordings. Using the manual input of anatomical references determined by an expert panel, parameters could be measured. 

## 2. Methods

### 2.1. Subject Populations

Patients with idiopathic Parkinson's disease (diagnosed by a neurologist) having subjective clinical complaints of dysphagia were recruited from several neurological departments in the Netherlands. All patients reported dysphagic complaints ranging from mild to severe, for example, these included slow eating, oral or pharyngeal passage disorder, coughing while drinking, and a diminished quality of life as a direct consequence of dysphagia. None of them suffered from any neurological disease except Parkinson's. All patients were able to perform a swallow. Other exclusion criteria were deep brain stimulation (DBS), a Mini Mental State Examination (MMSE) score below 23, severe dyskinesia of head and neck (resulting in problems with VFS recording), mental depression, head and neck cancer, severe cardiopulmological disease, speech therapy during the past six months, or surgery of the swallowing mechanism or the central nervous system [11]. All patients showed a stable disease period at the time of inclusion and had been on the same antiparkinsonian medication regimen for more than two months. The clinical severity of the disease was scored using the Hoehn and Yahr (H&Y) staging scale (Table 1) [12]. Healthy control subjects from the department of otorhinolaryngology, head and neck surgery of the university medical center were matched by age and gender. The age difference between the patient and his or her matched control subject was less than four years. The control subjects did not have complaints of swallowing, mental depression, any neurological disease, or head and neck cancer. Nor had they undergone surgery of the swallowing mechanism or the central nervous system. Written informed consent was obtained from all patients and healthy control subjects. This study protocol was approved by the medical ethical committee of the university medical center. 

### 2.2. Swallowing Assessment

All patients underwent a detailed clinical examination by an experienced laryngologist and a speech and language pathologist. Before the VFS examination the presence and severity of dysphagia were assessed using the Functional Oral Intake Scale (FOIS) and a fiberoptic endoscopic evaluation of swallowing (FEES) [13, 14]. Following these examinations, all subjects underwent a standardized videofluoroscopic swallowing protocol of three trials of thin liquid (forming part of a more elaborate experiment to be published in a subsequent report). Each trial contained 10 cc of low-density barium (40% w/v barium boluses) delivered orally by a syringe. The subjects had to swallow the bolus after it had been accurately delivered by syringe in the oral cavity resulting in a motor challenge without any preparatory cue. The VFS was performed in lateral position. Subjects were seated upright wearing their dental prosthesis (if present). The lips, oral cavity, cervical spine, and proximal cervical esophagus were included in the recording. A coin of five eurocents was fixed on the retroauricular skin as a reference distance to correct for magnification. Videofluoroscopic images were obtained with a Philips Diagnost 97 system (Philips Medical Systems, Eindhoven, The Netherlands) and recorded on cassette at 25 frames per second using a mini-DV camera-recorder Panasonic AG-DVC30 (Matsushita Electric Industrial Co., Osaka, Japan). If mild aspiration during a trial was observed, a subsequent trial was administered, but in case of severe aspiration the examination was ended. All examinations were performed within 90–120 minutes after the intake of antiparkinsonian medication, thus, during the “on” motor phase [15]. The VFS was performed by an independent experienced speech therapist together with the radiologist.

### 2.3. Measurements

Swallows were analyzed using a specific software application (Image & Physiology SL, Barcelona, Spain) to capture, digitize, and measure the videofluoroscopic swallowing sequences [16]. All variables are defined in Table 2.

Quantitative measurements were determined for each swallow by two experienced raters with extensive training in the analysis of VFS studies of normal and disordered swallowing. Kahrilas et al. used more physiologic references to calculate timed variables in contrast with fixed anatomical landmarks, like the point where the mandible crosses the tongue base [17]. In this study, timed variables for biomechanical analysis of swallowing were defined according to Kahrilas et al.: moment of opening and closing of the glossopalatal junction (GPJ); moment of opening and closing of velopharyngeal junction (VPJ); moment of opening and closing of laryngeal vestibule (LV); moment of opening and closing of upper esophageal sphincter (UES). The frame exhibiting penetration or aspiration was marked by the raters as timed event as well. Penetration was defined as passage of bolus into the laryngeal vestibule. Aspiration was defined as passage of bolus below the level of the vocal folds. 

**Figure 1:**
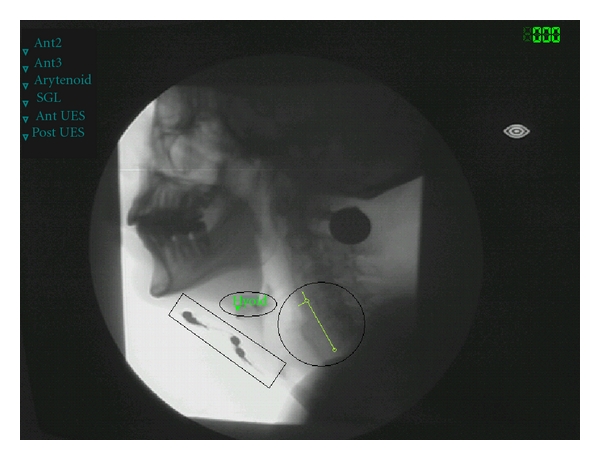
Single frame of the videofluoroscopic recording showing the landmarks used for spatial measurements. Extent of movement of the hyoid bone in the *x*-*y* coordinate system. The square encloses four electrodes in position during status “electrical current off,” the oval includes the marked hyoid bone, and the circle indicates the *x*-*y* coordinate system.

Movement patterns of the hyoid bone as described by Logemann et al. were used as spatial variables to analyze the swallowing function [18]. For each swallow three points were marked in each video frame: the anterior/superior corner of the hyoid bone and the anterior/inferior corner of the third and fifth cervical vertebral bodies. The *y*-axis was defined by the anterior/inferior corner of the third and fifth cervical vertebral bodies. The *x*-axis crosses the *y*-axis at the anterior/inferior corner of the third vertebral body. By marking these reference points in each frame, movements of the subject in any plane were corrected (Figure 1). Software analyzed the extent of movement of the hyoid bone in the *x*-*y* coordinate system over time. 

The following visuoperceptual parameters to evaluate videofluoroscopic signs were scored: preswallow anterior and preswallow posterior spill; lingual pumping; swallow hesitancy; piecemeal deglutition; delayed initiation of the pharyngeal reflex; postswallow oral residue; postswallow vallecular pooling; postswallow pyriform sinus pooling [7, 19]. Furthermore, the penetration aspiration scale according to Rosenbek et al. was scored for all VFS studies [20]. This eight-point scale (1–8) contains lower scores referring to normal functioning whereas higher scores refer to more severe disability. 

Following consensus training, two experts assessed all quantitative and qualitative variables independently at varying speed, ranging from normal to slow motion to frame-by-frame viewing. The raters were blinded to the diagnosis (Parkinson versus healthy subjects) and swallow trials were scored in randomized order. The consensus training in visuoperceptual evaluation was accompanied by a manual including strict, well-defined guidelines to rate these ordinal variables. The exact interpretation per level of each of the three or five point scales was trained during five separate sessions with intervals of one week. During these intervals the expert raters had to accomplish test trials separately that were discussed the next session. 

To obtain the intrarater reliability each rater performed repeated measurements of all temporal, spatial, and visuoperceptual variables in all swallows within a period of two weeks. 

### 2.4. Statistical Analysis

Statistical analysis was conducted stepwise. First, Intraclass Correlation Coefficients (ICC) and Cronbach's alphas (>0.65) were computed for all quantitative parameters to determine the reliability and the degree of intra- and interrater (absolute) agreement. For all ordinal parameters Cohen's Kappa index of agreement was used. Second, for each reliable temporal, spatial, and visuoperceptual variable, group comparisons were performed over the averaged data of the expert raters. Differences between the Parkinson and the healthy control populations were tested for significance by means of the Mann-Whitney *U* test (temporal and spatial, continuous variables) or the Chi-Square test (visuoperceptual, ordinal variables). All statistical analyses were performed using SPSS version 16.0 for Windows (SPSS Inc., Chicago, IL, USA). 

## 3. Results

### 3.1. Subject Populations

Ten mentally competent dysphagic patients (3 women, 7 men) with a diagnosis of idiopathic Parkinson's disease were included. The H&Y staging scale ranged from mild to moderate (median II). Table 1 shows the subject characteristics of all participants. The mean age of the patients and the controls was, respectively, 66 and 65 years. The duration of their Parkinson's disease ranged from 5 to 13 years. The median of the FOIS score in the Parkinson patients was 7. The range of scores of the FOIS is one to seven, indicating nothing by mouth to total oral diet with no restrictions. Subjective complaints of the patients included, for example, extremely slow eating, oral or pharyngeal passage disorder, food stuck in the throat, coughing while drinking, coughing during meals, and a diminished quality of life as a direct consequence of dysphagia.

### 3.2. Reliability of Quantitative and Qualitative Evaluation of Videofluoroscopy

In Tables 3(a), 3(b), and 3(c) the intrarater and interrater reliability of the applied variables is presented. Visuoperceptual variables like preswallow anterior and preswallow posterior spill, swallow hesitancy, and postswallow oral residue mainly scored zero points, meaning normal without any disturbances. It was decided to exclude these variables because of insufficient relevance to this Parkinson patient group. In contrast to the interrater reliability, it proved that the intrarater reliability was sufficient for some variables: delayed initiation pharyngeal reflex; UESd; GPJo-UESc; maximum horizontal (anterior) hyoid motion, for example. When the intrarater reliability proved to be rather low (ICC < 0.60), however, the interrater reliability was not computed; this was the case for GPJc, GPJd, UESo, GPJo-UESo, and penetration-aspiration scale, among others. 

### 3.3. Group Differences

Tables 4(a), 4(b), and 4(c) contain descriptive statistics of the reliable temporal, spatial, and visuoperceptual data (ICCs ≥ 0.60, Cohen's Kappa ≥ 0.60). To detect differences in swallowing physiology, data were tested for significant differences between Parkinson patients and the healthy control subjects. Using the Mann-Whitney *U* test, no significant differences were found regarding the spatial variables. The Chi-Square test did not reveal significant group differences for the visuoperceptual variables. The temporal variable VPJc showed a significant difference between the two groups. Patients with Parkinson's disease showed a significantly delayed velopharyngeal junction closure compared to healthy controls. 

## 4. Discussion

Before interpreting the present study outcome, the intrarater and interrater reliability was determined. Despite the thorough consensus training using well-defined guidelines as well as the strict methodological protocol for repeated measurements by the expert raters to calculate the intra- and interrater reliability in the present study poor reliability was observed for several parameters. Diverse situations may have contributed to this finding. First, despite their high expertise, the raters may have lacked consensus on a definition of, for example, delayed initiation of the pharyngeal reflex or maximum horizontal (anterior) hyoid motion. Second, patients were found to be rather homogeneous with respect to several of the variables being measured; that is, some of them, such as preswallow anterior spill and preswallow posterior spill, always scored zero. Finally, it cannot be excluded that the lack of internal consistency may have resulted from the nature of the measurement scales and/or instruments; for instance, visuoperceptual variables were scored on three or five point scales. As worldwide no strict guidelines exist, some scope for subjectivity was left to the rater in the discrimination of these ordinal variables. Consequently, these variables were less reliable compared to the temporal or spatial variables that were defined frame by frame using specific software [16]. This software-guided measurement technique, however, is not without its problems either. The fact that a few quantitative variables, for instance GPJo or UESo, were only present or visible in a single frame because of the high speed of movement during the swallowing act may have increased the measurement error and/or the rater's inaccuracy. Furthermore, the method proved to be rather labor intensive. Finally, fifteen variables could be used for further comparison between Parkinson patients and healthy control subjects. 

Despite the presence of a disturbed swallow physiology in Parkinson patients, few significant differences were found between the two pilot groups. Velopharyngeal junction closure (VPJc) was different, being significantly delayed in the Parkinson group. The remaining temporal, spatial, or visuoperceptual variables did not show significant differences or tendencies between the groups. The lack of significant group differences can be the result of the nature and sensitivity of the assessment tool (VFS). As described in the study of Ertekin et al. conflicting results obtained from radiological and manometric studies in Parkinson patients were found [21]. Also normal motility of the UES region during VFS was observed despite the presence of manometric abnormalities in the same region. In the present pilot study, preservation of the swallowing function during the early stages of Parkinson's disease was found using VFS despite the presence of subjective swallowing complaints in the patients. However, if other assessment tools, for example, manometry might have been used, the study outcome could have been different and abnormalities in swallowing may have been demonstrated. Ertekin et al. also described some compensatory mechanisms in the course of Parkinson's disease that may explain the benign nature of the deglutition disorder with preservation of swallowing until the terminal stage of the disease [21]. Other explanations for the lack of significance may lie in the advanced age of all subjects (presbyphagia), the rather small sample size, and the moderate severity of Parkinson's disease in the patient population (H&Y scale) [5, 7]. For several mainly logistic reasons (e.g., not being able to sit upright for VFS, too weak condition for repeated transport to the outpatient clinic for dysphagia, suffering from Parkinson dementia), patients with severe Parkinson disease who are often admitted to nursing homes did not find easy access to this study. However, the population of included patients was a realistic representation of Parkinson patients consulting a speech therapist for dysphagic complaints. Next, the mechanism of pharyngeal triggering is age dependent [22]. Probably, the natural aging process in the control subjects has been accompanied by altered swallowing function. Furthermore, the presence of electrodes on the skin, which had been used for another study during the same videofluoroscopic examination, may have worked as tactile cueing in the Parkinson group, even though no electrical stimulation was given [23]. Such cueing may have improved the swallowing performance. Finally, only low-density barium boluses were applied. The subjects had to swallow the bolus after it had been accurately delivered by syringe in the oral cavity. It is more difficult for patients with Parkinson's disease to initiate any movement from rest position rather than starting the movement from a previous cueing event or preparatory movement. Seeing this in the light of swallowing, initiating the oral phase is more difficult without any cue or previous movement like the labiobuccal movements or sensorial mucosal stimulation during drinking from a cup or chewing of solid foods than delivery of the bolus by syringe. So, it was decided to apply thin liquid boluses by syringe expecting the highest chance of observing deglutition disorders because of the motor challenge without any preparatory cue. However, other consistencies, such as high-density barium boluses or solid food consistencies, may lead to different dysphagic observations [24]. The discrepancy between symptoms of dysphagia in daily life and radiological results are common in Parkinson's disease, when comparing spontaneous behavior with swallowing under highly controlled experimental conditions [25]. Therefore, the question arises if the FOIS scale is a satisfactory measure for dysphagia severity in this patient population, given the normal scores in the present study.

In order to design therapy effect studies for dysphagia in Parkinson's disease, it is necessary to understand the pathophysiology of swallowing in this patient group. In previous studies, several authors investigated pathological mechanisms of oral-pharyngeal dysphagia in Parkinson patients [9, 19]. Several authors suggested rigidity, hypokinesia, and bradykinesia as factors underlying the disordered oral and pharyngeal stages of swallowing [6, 9]. Also incomplete cricopharyngeal relaxation, reduced cricopharyngeal opening, and delayed initiation of the swallowing reflex have been suggested as possible mechanisms of dysphagia in this population [9, 19]. The authors indicate that parkinsonian patients may be “silent aspirators” with decreased cough reflexes and lack of awareness of aspiration. Abnormal findings in the oral and pharyngeal phase of swallowing were described based on temporal and visuoperceptual variables. Nagaya et al. observed that durational changes in the pharyngeal phase of swallowing in Parkinson patients were similar to natural changes in elderly control subjects [5]. In the present study differences in swallowing physiology between Parkinson patients and age- and gender-matched healthy control subjects were investigated using qualitative and quantitative variables. These variables to evaluate the oropharyngeal swallowing function in Parkinson's disease were obtained from the very few existing studies on this matter [5, 6, 9, 19]. These studies showed group differences between Parkinson patients and healthy control subjects. However, methodological shortcomings were found in all studies, for example, measurements in the “off” motor phase of Parkinson's disease, only one rater, no consensus training in case of more raters, no blinding, no information on the protocol of repeated measurements, neither on the rating scale of the ordinal variables. So far, the evidence of these studies is neither strong nor reliable enough to serve as comparison for the present study. Despite diverse methodological problems, these studies provided interesting suggestions for variables that could be used in the present study. 

In this pilot study, few restrictions were placed on the kind and number of parameters to use. Per swallow (*N* = 60 swallows in total) thirty variables were assessed, resulting in 1800 measurements. The selection of parameters in follow-up studies should be based on sufficient intra- and interrater reliability as well as on a patient data distribution using the full range of the parameters' scale. The literature on swallowing assessment usually provides only limited information on the definitions of the parameters being used, the exact description of the protocol applied during videofluoroscopy, the number of raters or the explanation of the concept of an “expert” or “experienced” rater, the intra- and interrater reliability, and the effect of training on reliability coefficients. Interpretation of any study outcome lacking such information may be hazardous. Dysphagic Parkinson patients could potentially benefit from improved diagnostic swallowing assessment by implementing well-defined videofluoroscopic parameters with sufficient intra- and interrater reliability.

## 5. Conclusions

Despite the special attention paid to methodology in this present study, insufficient reliability for fifteen out of thirty variables (temporal, spatial, and visuoperceptual) was found, particularly for the visuoperceptual variables. The reliable variables could only reveal very few significant differences between both pilot groups. Swallowing seems relatively preserved in the early stages of Parkinson's disease. Recommendations for future research on pathophysiological aspects of swallowing in Parkinson's disease may include study designs with larger numbers of dysphagic patients and healthy control subjects. It may be interesting too to include patients with a more severe degree of Parkinson's disease (higher scores on the Hoehn and Yahr scale) to determine differences between such a patient population and the patients who visit speech therapists as described in this study. The present study has found relatively low intrarater and interrater reliability for many of the variables used despite thorough training and high level of the raters' expertise. In the literature, information on reliability is usually lacking. After training, raters should have maximum consensus about the exact definition of the variables included and be familiar with the rating scales being used as well as with their levels, preferably anchored in detailed descriptions. Any swallowing study should provide information on training and the intrarater and the interrater reliability in order to allow accurate interpretation of the study outcome. Maybe the suggestion that data described in earlier studies may lack sufficient reliability and, therefore, may not be useful in determining therapy outcome, has been rather unexpected. Still, the problems with reliability in outcome studies such as described in this paper may also be the most interesting as well as the most important finding. Newly developed research will need to take this issue into account so that in the near future, in research on the physiology of swallowing in Parkinson's disease, the complementary benefits of using qualitative as well as quantitative variables in videofluoroscopy can be studied more thoroughly. 

## Figures and Tables

**Table 1:** Subject characteristics.

Number of matched pair of subjects	Sex (female/male)	Patients with Parkinson's disease				Healthy controls
		Hoehn and Yahr scale^a^ (H&Y)	Duration of Parkinson's disease (years)	Functional oral intake scale^b^ (FOIS)	Age (years)	Age (years)
1	M	I	5	7	70	68
2	F	II	7	7	64	63
3	M	II	7	7	50	46
4	M	III	6	7	80	81
5	F	II	>5	7	73	74
6	M	III	5	7	57	53
7	F	III	7	7	62	60
8	M	II	7	5	70	67
9	M	III	16	7	70	70
10	M	I	>5	7	66	68

^a^(H&Y): the range of scores is one to five, indicating, respectively, unilateral involvement usually with minimal or no functional disability, and confinement to bed or wheelchair unless aided [12].

^b^(FOIS): the range of scores is one to seven, indicating nothing by mouth to total oral diet with no restrictions [13].

**Table 2:** Measurements in videofluoroscopy.

Method of measurement	Parameters		
	Name	Definition	Rating scale or units
Temporal parameters	GPJo (Glossopalatal junction opening)	Moment of separation of the tongue and soft palate.	Seconds
	GPJc (Glossopalatal junction closure)	Moment of contact of the tongue and palate after bolus propulsion.	
	GPJd (Glossopalatal junction duration)	ΔT between GPJo and GPJc.	
	VPJc (Velopharyngeal junction closure)	Moment of first contact of the soft palate against the posterior pharyngeal wall.	
	VPJo (Velopharyngeal junction opening)	Moment of separation of the soft palate and the posterior pharyngeal wall with reentry of air in the retrolingual space from the nasopharynx.	
	VPJd (Velopharyngeal junction duration)	ΔT between VPJc and VPJo	
	LVc (Laryngeal vestibule closure)	Moment when laryngeal elevation results in making contact between the arytenoid cartilages and the underside of the epiglottis.	
	LVo (Laryngeal vestibule opening)	Moment of separation of the arytenoid cartilages and the underside of the epiglottis with reentry of air in the laryngeal vestibule.	
	LVd (Laryngeal vestibule duration)	ΔT between LVc and LVo	
	UESo (Upper esophageal sphincter opening)	Moment of opening of the esophagus with entry of either air or barium.	
	UESc (Upper esophageal sphincter closure)	Moment of closure of the esophagus after bolus transport.	
	UESd (Upper esophageal sphincter duration)	ΔT between UESo and UESc	
	GPJo-LVc	ΔT between GPJo and LVc	
	GPJo-UESo	ΔT between GPJo and UESo	
	GPJo-UESc	ΔT between GPJo and UESc	
	Aspiration-penetration	Moment of aspiration or penetration	

Spatial and temporal parameters (hyoid motion)	Horizontal hyoid motion	Maximum horizontal (anterior) motion during swallowing act.	Millimeters
	Vertical hyoid motion	Maximum vertical motion during swallowing act.	Millimeters
	Duration vertical hyoid motion	Duration between initiation of swallow and moment of maximum vertical motion.	Seconds
	Duration horizontal hyoid motion	Duration between initiation of swallow and moment of maximum horizontal (anterior) motion.	Seconds

Visuoperceptual parameters^a^	Preswallow anterior spill	Preswallow loss of bolus from the lips	Five-point scale (0–4)
	Preswallow posterior spill	Preswallow loss of bolus into the pharynx	Five-point scale (0–4)
	Lingual pumping	Preswallow involuntary repetitive tongue movements	Five-point scale (0–4)
	Swallow hesitancy	Delayed onset oral transport	Three-point scale (0–2)
	Piecemeal deglutition	Sequential swallowing on the same bolus	Five-point scale (0–4)
	Delayed initiation pharyngeal reflex	Delayed onset pharyngeal triggering	Three-point scale (0–2)
	Postswallow oral residue	Postswallow pooling in the oral cavity	Five-point scale (0–4)
	Postswallow vallecular pooling	Postswallow pooling in the valleculae	Three-point scale (0–2)
	Postswallow pyriform sinus pooling	Postswallow pooling in the pyriform sinuses	Three-point scale (0–2)
	Penetration aspiration scale (PAS) [20]	Penetration and/or aspiration	Eight-point scale (1–8)

^a^Lower scores refer to normal functioning whereas higher scores refer to more severe disability.

**Table:** (a)

Temporal variable	Intrarater test-retest reliability (ICC)		Interrater reliability (ICC)
	Rater 1	Rater 2	
GPJ opening	—	—	—
GPJ closure	0.4	0.8	—
GPJ duration	0.4	0.8	—
VPJ closure	0.6	0.8	0.7
VPJ opening	1.0	1.0	0.7
VPJ duration	0.9	1.0	0.7
LV closure	0.9	0.9	0.9
LV opening	0.9	0.6	1.0
LV duration	1.0	0.7	0.9
UES opening	0.4	0.5	—
UES closure	0.6	0.8	0.8
UES duration	0.7	0.8	−0.1
GPJo_LVc	0.9	0.9	0.9
GPJo_UESo	0.4	0.9	—
GPJo_UESc	0.6	0.8	−0.02
Aspiration or penetration	—	—	—

^a^Single measures ICC, Cronbach's alpha >0.65.

**Table:** (b)

Spatial-temporal variable	Intrarater test-retest reliability (ICC)		Interrater reliability (ICC)
	Rater 1	Rater 2	

Maximum horizontal (anterior) hyoid motion.	0.9	0.6	—
Maximum vertical hyoid motion.	0.8	0.9	0.8
Duration between initiation of swallow and moment of maximum horizontal (anterior) hyoid motion.	0.9	0.9	0.9
Duration between initiation of swallow and moment of maximum vertical hyoid motion.	0.6	0.7	0.9

^a^Single measures ICC, Cronbach's alpha >0.65.

**Table:** (c)

Visuoperceptual variable	Intrarater test-retest reliability		Interrater reliability
	Cohen's Kappa		Cohen's Kappa
	Rater 1	Rater 2	Rater 1-2

Preswallow anterior spill	—	—	—
Preswallow posterior spill	1.0	1.0	—
Lingual pumping	1.0	1.0	0.6
Swallow hesitancy	1.0	—	—
Piecemeal deglutition	1.0	1.0	0.9
Delayed initiation pharyngeal reflex	0.8	1.0	0.3
Postswallow oral residue	0.6	0.7	—
Postswallow vallecular pooling	0.9	0.9	0.6
Postswallow pyriform sinus pooling	0.8	1.0	0.6
Penetration aspiration [20]	1.0	—	—

**Table:** (a)

Temporal parameters	Parkinson's disease		Healthy controls		Statistical analyses
	Median	25′, 75′ perc.	Median	25′, 75′ perc.	*P* value
VPJ closure	0.08	0.02, 0.12	0.03	−0.02, 0.06	0.02
VPJ opening	0.76	0.64, 1.00	0.71	0.66, 0.78	0.31
VPJ duration	0.70	0.55, 0.94	0.66	0.64, 0.74	0.78
LV closure	0.13	0.10, 0.26	0.12	0.10, 0.20	0.58
LV opening	0.91	0.72, 0.98	0.84	0.78, 0.91	0.51
LV duration	0.69	0.60, 0.80	0.66	0.60, 0.78	0.78
UES closure	0.86	0.74, 1.14	0.79	0.74, 0.86	0.13
GPJo-LV closure Δ*t* (sec)	0.13	0.10, 0.26	0.12	0.10, 0.20	0.58

**Table:** (b)

Spatial-temporal parameters	Parkinson's disease		Healthy controls		Statistical analyses
	Median	25′, 75′ perc.	Median	25′, 75′ perc.	*P* value

Maximum vertical hyoid motion (mm)	13.68	9.26, 20.02	14.68	13.26, 19.50	0.35
Duration between initiation of swallow and moment of maximum anterior/horizontal hyoid motion Δ*t* (sec)	1.64	1.01, 2.62	1.12	0.86, 2.16	0.29
Duration between initiation of swallow and moment of maximum vertical hyoid motion Δ*t* (sec)	1.72	1.04, 2.09	1.28	0.88, 2.04	0.48

**Table:** (c)

Visuoperceptual (ordinal) parameters	Parkinson's disease							Healthy controls						*P* value

Lingual pumping	0	1		2	3	4		0	1		2	3	4	0.35
	76%	17%		7%	0%	0%		83%	17%		0%	0%	0%	
	(23)	(5)		(2)	(0)	(0)		(25)	(5)		(0)	(0)	(0)	

Piecemeal deglutition	0	1	2	3	4			0	1	2	3	4		0.23
	31%	41%	24%	4%	0%			39%	54%	7%	0%	0%		
	(9)	(12)	(7)	(1)	(0)			(11)	(15)	(2)	(0)	(0)		

Postswallow vallecular pooling	0	1		2				0	1		2			0.16
	48%	42%		10%				45%	55%		0%			
	(14)	(12)		(3)				(13)	(16)		(0)			

Postswallow pyriform sinus pooling	0	1		2				0	1		2			0.06
	83%	17%		0%				59%	41%		0%			
	(25)	(5)		(0)				(17)	(12)		(0)			
